# COVID-19 Vaccine Reactogenicity Among Young Children

**DOI:** 10.1001/jamanetworkopen.2024.47492

**Published:** 2024-11-25

**Authors:** Sabrina A. Madni, Kieauna Strickland, Victoria Konrad, Lauren Head Zauche, Christine K. Olson, Andrea J. Sharma

**Affiliations:** 1 Division of Healthcare Quality Promotion, National Center for Emerging and Zoonotic Infectious Diseases, Centers for Disease Control and Prevention, Atlanta, Georgia; 2 Deloitte Consulting LLP, Atlanta, Georgia; 3 Division of Birth Defects and Infant Disorders, National Center on Birth Defects and Developmental Disabilities, Centers for Disease Control and Prevention, Atlanta, Georgia; 4 GenTech Associates, Inc, Zionsville, Indiana; 5 US Public Health Service Commissioned Corps, Atlanta, Georgia

## Abstract

This cross-sectional study examines reactogenicity among children of participants in the Centers for Disease Control and Prevention COVID-19 Vaccine Pregnancy Registry from November 2022 to September 2023 to understand the frequency and types of reactions experienced.

## Introduction

COVID-19 vaccine reactogenicity data among young children, which inform parental decisions and health care professional recommendations for vaccination, are limited. Reactogenicity can be described as expected and common symptoms after vaccination (eg, pain at injection site). We assessed reactions to COVID-19 vaccines among children of participants in the Centers for Disease Control and Prevention (CDC) COVID-19 Vaccine Pregnancy Registry (C19VPR).

## Methods

From November 2022 to September 2023, C19VPR participants (people receiving a COVID-19 vaccine 30 days before last menstrual period or during pregnancy) who reported a live birth were asked at least 15 months after delivery about their child’s COVID-19 vaccination status. We asked participants to report local and systemic reactions, severity of reactions, reaction-related health care, and coadministration of other vaccines for each COVID-19 vaccine their child received; data were collected for up to 3 vaccinations. This activity was reviewed by the CDC and deemed public health surveillance, not research; it was exempt from Human Research Protections Office review and was conducted consistent with applicable federal law and CDC policy—for example, see, eg, 45 CFR part 46.102(l)(2), 21 CFR part 56; 42 USC §241(d); 5 USC §552a; 44 USC §3501 et seq. Details on C19VPR methodology are available^1^; additional analytic details are available in eMethods in Supplement 1. We excluded children without information on vaccine manufacturer and/or date. Participant race and ethnicity were self-reported and classified into smaller groups based on CDC methods for grouping race and ethnicity.^2^

We compared prevalence of adverse reactions by vaccine manufacturer, dose, and coadministration, using prevalence ratios adjusted for variables associated with reactions (ie, child age at dose 1 and history of childhood SARS-CoV-2 infection). Reactions across all doses were compared by manufacturer using binomial regression with generalized estimating equations to account for correlations introduced by including more than 1 dose for the same child. This study followed the STROBE reporting guideline. Analysis was conducted in SAS, version 9.4 (SAS Institute). Statistical tests were 2-sided with significance set as *P* < .05.

## Results

Among 5644 children who received at least 1 dose of COVID-19 vaccine (2569 [45.5%] BNT162b2 [Pfizer-BioNTech]; 2972 [52.7%] mRNA-1273 [Moderna]), the mean age at first dose was 12.4 months (minimum, 6 months; maximum, 24 months) (Table; Figure). Coadministration of dose 1 with another vaccine was reported for 20.0% of children. Postvaccination reactions were reported for 46.7% of children, with 21.1% and 38.8% experiencing local and systemic reactions, respectively. Commonly reported reactions included irritability or fussiness (1700 [30.1%]), local reaction (1191 [21.1%]), and fever (779 [13.8%]). Among children with reported reactions, 18 of 2633 (0.7%) experienced reactions described by participants as severe. In total, 87 of 5644 children (1.5%) were reported to have received care from a health care professional for a reaction after any dose (BNT162b2, 45 [1.8%]; mRNA-1273, 42 [1.4%]). Six participants reported a seizure or febrile seizure after receiving COVID-19 vaccination. No deaths were reported.

**Table.** Data on Adverse Reactions After COVID-19 Vaccination Among Young Children, by Vaccine Manufacturer and by Coadministration Status at Dose 1, Centers for Disease Control and Prevention COVID-19 Vaccine Pregnancy Registry, November 2022 to September 2023^a^

Data	Total, No. (%)	Manufacturer of COVID-19 vaccine received^b^			Coadministration at dose 1 of COVID-19 vaccine^c^		
		No. (column %)		APR (95% CI)	No. (column %)		APR (95% CI)
		BNT162b2 only	mRNA-1273 only		Yes	No	
No. (row %)	5644 (100)	2569 (45.5)	2972 (52.7)	NA	1081 (20.0)	4313 (80.0)	NA
Any reaction^d^	2633 (46.7)	1164 (45.3)	1409 (47.4)	1.14 (1.09-1.20)	537 (49.7)	1647 (39.2)	1.20 (1.13-1.29)
Any local reaction^e^	1191 (21.1)	504 (19.6)	658 (22.1)	1.42 (1.26-1.61)	276 (25.5)	71 (16.6)	1.47 (1.31-1.67)
Any systemic reaction	2189 (38.8)	968 (37.7)	1168 (39.3)	1.22 (1.11-1.33)	423 (39.1)	1316 (30.5)	1.16 (1.07-1.26)
Fever	779 (13.8)	330 (12.9)	427 (14.4)	1.28 (1.13-1.44)	146 (13.5)	392 (9.1)	1.23 (1.05-1.46)
Irritability or fussiness	1700 (30.1)	748 (29.1)	910 (30.6)	1.08 (1.01-1.16)	341 (31.5)	997 (23.1)	1.20 (1.09-1.33)
Loss of appetite	205 (3.6)	94 (3.7)	102 (3.4)	1.13 (0.88-1.46)	45 (4.2)	81 (1.9)	1.42 (1.03-1.96)
Vomiting	41 (0.7)	20 (0.8)	19 (0.6)	0.79 (0.43-1.46)	3 (0.3)	15 (0.4)	0.43 (0.15-1.24)
Diarrhea	70 (1.2)	38 (1.5)	31 (1.0)	0.95 (0.62-1.45)	13 (1.2)	33 (0.8)	1.28 (0.72-2.30)
Rash not around injection site	93 (1.7)	45 (1.8)	47 (1.6)	1.08 (0.73-1.60)	19 (1.8)	43 (1.0)	1.35 (0.82-2.22)
Fatigue or lethargy	314 (5.6)	144 (5.6)	166 (5.6)	1.04 (0.87-1.25)	44 (4.1)	184 (4.3)	1.05 (0.79-1.40)
Other systemic reaction	40 (0.7)	14 (0.5)	23 (0.8)	2.23 (1.12-4.42)	6 (0.6)	15 (0.4)	0.70 (0.27-1.83)

Abbreviations: APR, adjusted prevalence ratio; NA, not applicable.

^a^ Prevalence ratios are adjusted for child’s age at dose 1 and history of childhood SARS-CoV-2 infection, which were associated with reactions based on χ^2^ analyses. Referent groups were BNT162b2 and no coadministration.

^b^ Participants were asked to provide details for up to 3 child COVID-19 vaccinations. Median time from dose 1 to interview was 6.9 months (IQR, 5.0-8.7 months). Of the total vaccinated, 103 children (1.8%) received second or third COVID-19 vaccine doses from a manufacturer different from their first dose. Among children who received a first dose of BNT162b2, 29 received their second or third dose of mRNA-1273 or an unknown manufacturer and were excluded from the BNT162b2 column. Among children who received a first dose of mRNA-1273, 74 received their second or third dose of BNT162b2 or an unknown manufacturer and were excluded from the mRNA-1273 column.

^c^ Received at least 1 other routine childhood vaccine at the same time as COVID-19 vaccine. Of the total, 250 participants reported being unsure whether their child received additional routine childhood vaccines at the same time as their first dose of COVID-19 vaccine and were excluded from coadministration analyses.

^d^ Reaction is coded as reaction reported at any dose. Local and systemic reaction and their subtypes are not mutually exclusive.

^e^ Any local reaction included pain, redness, swelling, rash, or swollen lymph node.

**Figure.**
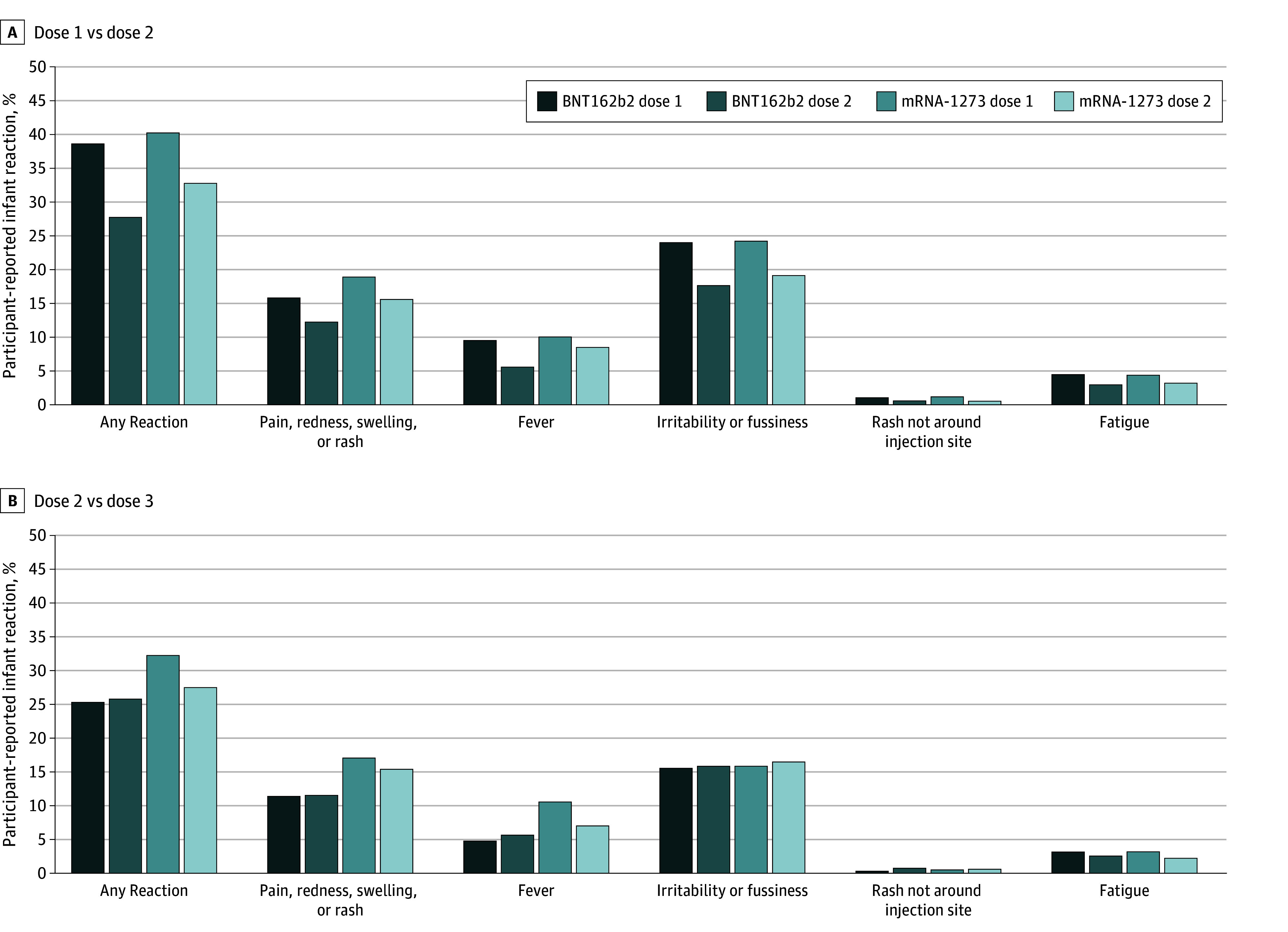
Percentage of Reactions Among Young Children With Known Vaccine Manufacturer Who Received at Least 1 Dose of COVID-19 Vaccine by Manufacturer and Dose Number, Centers for Disease Control and Prevention COVID-19 Vaccine Pregnancy Registry, November 2022 to September 2023 Children who received doses 1 and 2 from the same manufacturer were included in the denominator for dose 1 vs 2 comparisons (BNT162b2, 2376; mRNA-1273, 2865). Children who received doses 2 and 3 from the same manufacturer were included in the denominator for dose 2 vs dose 3 comparisons (BNT162b2, 1682; mRNA-1273, 630). The McNemar test for paired proportions was used to examine differences in reactions between doses 1 and 2 and doses 2 and 3 by manufacturer. Within each manufacturer group, significant differences (*P* < .05) between doses 1 and 2 were identified for all displayed reactions, except rash not around injection site for BNT162b2 (*P* = .80). For doses 2 and 3, only any reaction and fever were significantly different for children receiving mRNA-1273 vaccines; there were no significant differences between doses 2 and 3 for children receiving only BNT162b2 vaccines.

Among children receiving doses from the same manufacturer, a report of any reaction was significantly higher after dose 1 than dose 2 (BNT162b2, 39.1% vs 28.1%; mRNA-1273, 40.7% vs 33.1%). Any reaction was more common among children receiving only mRNA-1273 compared with only BNT162b2 (adjusted prevalence ratio, 1.14; 95% CI, 1.09-1.20) and with coadministration of another vaccine (adjusted prevalence ratio, 1.20; 95% CI, 1.13-1.29).

## Discussion

No unexpected reactions were identified. Similar to our study, data from clinical trials and V-safe found that irritability was the most common systemic reaction among children aged 6 months to younger than 2 years, followed by fever and fatigue or sleepiness.^3,4,5,6,7^ In contrast to other studies observing a higher prevalence of reactions after the second COVID-19 vaccine dose, we observed a higher prevalence after the first dose. This difference may reflect maternal vaccination; the first COVID-19 vaccination in our cohort may be some children’s second immunological encounter with a COVID-19 vaccine. Generalizability may be limited because of a relatively demographically homogenous cohort, and participant-reported data may be subject to recall bias. These findings add evidence indicating that mild or moderate local and systemic reactions may be experienced, but severe reactions and serious adverse events are rare.

## Notes

Supplement 1.
eMethods.

Supplement 2.
Data Sharing Statement
