# Error in Results

**DOI:** 10.1001/jamanetworkopen.2024.58339

**Published:** 2025-01-06

In the Research Letter titled “COVID-19 Vaccine Reactogenicity Among Young Children,”^1^ published November 25, 2024, there was an error in the fifth sentence of the first paragraph of the Results section; among children with reported reactions, 18 of 2633 (0.7%) experienced reactions described by participants as severe, not 18 of 56 (0.3%). This article has been corrected.^1^
