# Supplementary material for: COVID-19 Vaccine Reactogenicity Among Young Children
Source: JAMA Netw Open. 2024 Nov 25;7(11):e2447492. doi: 10.1001/jamanetworkopen.2024.47492 (PMC11589793; doi:10.1001/jamanetworkopen.2024.47492)
Supplement: Supplement 1. — eMethods. [file jamanetwopen-e2447492-s001.pdf]

Supplemental Online Content

Madni SA, Strickland K, Konrad V, Zauche LH, Olson CK, Sharma AJ. COVID-19 vaccine reactogenicity among young children. *JAMA Netw Open*. 2024;7(11):e2447492. doi:10.1001/jamanetworkopen.2024.47492

eMethods.

This supplemental material has been provided by the authors to give readers additional information about their work.

© 2024 Madni SA et al. *JAMA Network Open*.

eMethods.

CDC COVID-19 Vaccine Pregnancy Registry (C19VPR) Study Design

In Phase 1 of the C19VPR (conducted January 2021 – August 2022), people who 1) reported into V-safe that they received of a COVID-19 vaccination from December 2020 – June 2021, and 2) received at least one COVID-19 vaccine within 30 days prior to their last menstrual period (LMP) or during pregnancy were enrolled and completed up to six surveys; a detailed description of eligibility and methodology is available elsewhere.1 In Phase 2 (conducted November 2022 – September 2023), eligible participants were re-contacted to complete a cross-sectional survey at least 15 months after participants' pregnancy end dates. Participants contacted for Phase 2 participants include those not lost to follow-up in Phase 1 who consented to additional contact, regardless of pregnancy outcome. Phase 2 data collection included question on postpartum health and, among participants reporting a live birth, child health.

When COVID-19 vaccines were approved in June 2022 for children ≥6 months, 98% of children in the registry were ≥6 months of age. Since participants were called after their child had reached at least 15 months of life, all children were eligible for COVID-19 vaccination by the time of data collection. Data were collected for 9,166 children during Phase 2. Participants' children were excluded from this analysis if: the pregnancy occurred via surrogacy (n=9); the child died prior to 6 months of age (n=16); the participant did not report child COVID-19 vaccination status (n=23); COVID-19 vaccination date was unknown (n=122) or reported to have occurred prior to 6 months of age (n=17); or dose 1 vaccine manufacturer was unknown or reported to be from a manufacturer other than Pfizer-BioNTech or Moderna (n=168). Exclusions resulted in a final sample size of 8,811 children, 5,644 of whom were reported to have received at least one COVID-19 vaccine.

Phase 2 included questions about demographic characteristics, participant health, and child health and COVID-19 vaccination status (among participants reporting a live birth). Data on

© 2024 Madni SA et al. *JAMA Network Open*.

participants' children born prior to or after the pregnancy of interest (index pregnancy) were not collected. Participants were asked about their child's COVID-19 vaccination status and, if vaccinated, about vaccine manufacturer, administration date, local and systemic reactions, and medical care received after vaccination for up to three doses of COVID-19 vaccine. Participants were also asked whether their child received any other childhood vaccines at the same time as their COVID-19 vaccine; however, specification of other vaccines received was not ascertained. Participants were asked to indicate to whether their child experienced any of the following reactions: (1) pain, redness, swelling, or rash at injection site, (2) fever (no specified temperature threshold), (3) irritability or fussiness, (4) loss of appetite, (5) vomiting, (6) diarrhea, and (7) rash not around the injection site. Participants were also asked to report whether their child experienced any other reaction and, if so, to describe the reaction. Participants reported their perception of the severity of their child's reaction (mild, moderate, or severe) and whether their child received care from a doctor or other healthcare professional for symptoms related to the COVID-19 vaccine.

Outcomes

Survey questions about local and systemic reactions were used to assess prevalence of reactions. Broad categories were created for "any reaction," "any local reaction," and "any systemic reaction;" "pain, redness, swelling, or rash at injection site" was included in "any local reaction" and all other reaction categories were included as "any systemic reaction." Other specified reactions were reviewed and, where appropriate, recoded into one of the aforementioned reaction categories or into a new post-hoc category due to frequent reporting. This review resulted in the creation of a "fatigue or lethargy" category. Lymphadenopathy or swollen lymph node was coded as "any local reaction." All other remaining specified reactions were deemed as "other systemic reaction" and were included in the "any systemic reaction" category.

Covariates

© 2024 Madni SA et al. *JAMA Network Open*.

Child age at dose 1 was calculated as a continuous variable from participant-reported date of first COVID-19 vaccine and date of birth. History of COVID-19 infection was categorical with options of “yes,” “no,” and “unknown.” Participants could report COVID-19 diagnosis based on positive COVID-19 test or symptoms and/or exposure to a known COVID-19 case.

Modeling

We estimated prevalence ratios for each reaction by manufacturer using binomial logistic regression using Proc Genmod. Because each child could have received up to 3 doses, we accounted for repeated measures by including subject ID in the repeated statement and specifying an exchangeable correlation structure.”

© 2024 Madni SA et al. *JAMA Network Open*.
