# Supplementary material for: COVID-19 Vaccine Reactogenicity Among Young Children
Source: JAMA Netw Open. 2024 Nov 25;7(11):e2447492. doi: 10.1001/jamanetworkopen.2024.47492 (PMC11589793; doi:10.1001/jamanetworkopen.2024.47492)
Supplement: Supplement 2. — Data Sharing Statement [file jamanetwopen-e2447492-s002.pdf]

Data Sharing Statement

Madni. COVID-19 Vaccine Reactogenicity Among Young Children. *JAMA Netw Open*. Published November 25, 2024. doi:10.1001/jamanetworkopen.2024.47492

Data
Data available: No

Additional Information
Explanation for why data not available: Once all variables have been cleaned, a clean dataset may be made publicly available.
